# Design and Optimization of Sesamol Nanosuspensions to Potentiate the Anti-Tumor Activity of Epirubicin against Ehrlich Solid Carcinoma-Bearing Mice

**DOI:** 10.3390/pharmaceutics16070937

**Published:** 2024-07-13

**Authors:** Kholoud A. Elzanaty, Gamal A. Omran, Ehab Kotb Elmahallawy, Ashraf Albrakati, Ayman A. Saleh, Naief Dahran, Alaa S. Alhegaili, Ahmad Salahuddin, Heba Abd-El-Azim, Ahmed Noreldin, Tarek M. Okda

**Affiliations:** 1 Department of Biochemistry, Faculty of Pharmacy, Damanhour University, Damanhour 22511, Egypttarekokda@pharm.dmu.edu.eg (T.M.O.); 2 Grupo de Investigación en Sanidad Animal y Zoonosis (GISAZ), Departamento de Sanidad Animal, Universidad de Córdoba, 14071 Córdoba, Spain; 3 Department of Zoonoses, Faculty of Veterinary Medicine, Sohag University, Sohag 82524, Egypt; 4 Department of Human Anatomy, College of Medicine, Taif University, P.O. Box 11099, Taif 21944, Saudi Arabia; a.albrakati@tu.edu.sa; 5 Department of Pathology, College of Medicine, University of Hail, Hail 55428, Saudi Arabia; aym.ali@uoh.edu.sa; 6 Department of Anatomy, Faculty of Medicine, University of Jeddah, Jeddah 21959, Saudi Arabia; 7 Department of Medical Laboratory, College of Applied Medical Sciences, Prince Sattam bin Abdulaziz University, Al-Kharj 11942, Saudi Arabia; 8 Department of Biochemistry, College of Pharmacy, Al-Ayen Iraqi University, Nasiriyah 64001, Iraq; 9 Department of Pharmaceutics, Faculty of Pharmacy, Damanhour University, Damanhour 22511, Egypt; heba.mohamed@pharm.dmu.edu.eg; 10 Division of Engineering in Medicine, Department of Medicine, Brigham and Women’s Hospital, Harvard Medical School, Boston, MA 02115, USA; 11 Department of Histology and Cytology, Faculty of Veterinary Medicine, Damanhour University, Damanhour 22511, Egypt

**Keywords:** breast cancer, Ehrlich solid tumor, sesamol, Epirubicin, angiogenesis, apoptosis, autophagy

## Abstract

There is a growing interest in discovering natural sources of anti-cancer drugs. Sesamol (SES) is a phenolic compound with antitumor effects. The present study aimed to investigate the anticancer properties of SES and its nano-suspensions (SES-NS) combined with Epirubicin (EPI) in breast cancer (BC) using mice bearing a solid Ehrlich tumor. The study involved 35 female albino mice and investigated the effects of SES and EPI on tumor growth, proliferation, apoptosis, autophagy, angiogenesis, and oxidative stress. Methods including ELISA, qRT-PCR, and immunohistochemistry were utilized. The findings revealed reductions in tumor growth and proliferation using SES either alone or combined and evidenced by decreased AKT (AKT Serine/Threonine kinase1) levels, angiogenesis indicated by lower levels of VEGFR (vascular endothelial growth factor), and apoptosis demonstrated by elevated caspase3 and BAX levels. Furthermore, autophagy increased and was indicated by increased levels of beclin1 and lc3, along with decreased oxidative stress as evidenced by elevated TAC (total antioxidant capacity) and reduced MDA (malondialdehyde) levels. Interestingly, SES-NS demonstrated more significant effects at lower doses. In summary, this study underscores the potential of SES as a promising agent for BC treatment. Moreover, SES-NS potentiated the beneficial effects of EPI while mitigating its adverse effects.

## 1. Introduction

Breast cancer (BC) is considered one of the most prevalent types of cancer with more than 2.26 million cases diagnosed globally in 2020 [1]. Currently, BC accounts for 1 in 8 cancer cases, surpassing lung cancer as the most frequent cancer diagnosed globally [2]. The administration of high doses of chemotherapeutic medications in breast cancer treatment often leads to harmful side effects and the development of drug resistance [3]. Hence, it is crucial to discover and identify more potent medications with fewer adverse effects [4]. Among others, Epirubicin (EPI) is categorized as an anthracycline antitumor drug, exerting its effect through intercalating into DNA strands or binding to DNA topoisomerase II. This action blocks DNA and RNA synthesis, ultimately hindering tumor cell proliferation [5]. Numerous cancers, including ovarian, gastric, bladder, breast, and multiple myelomas, have been successfully treated with EPI [6,7]. It is frequently utilized in the treatment of BC with a higher survival rate [8]. Despite its efficacy approval, several side effects and cumulative dose-related cardiotoxicity has limited its clinical use [9].

It should be noted that the Ehrlich solid tumor (EST) is an in vivo experimental model employed in the study of BC in animals [10]. It was used successfully to investigate the formation and progression of tumors and the antitumor properties of natural products, nanoparticles, and synthetic chemicals [11]. Interestingly, natural products are compounds commonly employed to raise the therapeutic index of chemotherapy medications [12]. These compounds serve as valuable resources for the discovery and development of anticancer medications due to their non-toxic nature, efficacy against various tumor types, and lack of resistance [13].

Sesamol (SES) is a naturally occurring phenolic compound derived from sesame seeds (*Sesamum indicum*) [14]. SES has been extensively studied for its various biological effects, including inhibition of lipid peroxidation, enhanced radical scavenging [15], modulation of gene expression related to apoptosis such as caspase-3, BAX, Bcl2, and p53, and triggering apoptosis and stopping the cell cycle at certain points [16]. When examining the apoptotic and inflammatory pathways in thyroid cancer, Bcl-2 and BAX levels serve as crucial markers [17]. Nanomedicines offer substantial evidence to enhance the therapeutic effectiveness of formulated drugs by improving solubility, absorption, penetration, bioavailability, targeted delivery, physicochemical stability, dosage reduction, and pharmacological effect enhancement [18]. Phospholipid vesicles, a recent addition to pharmaceutical formulations, are specifically designed to enhance solubility [19]. Over the years, pharmaceuticals formulated in nanostructures have shown enhanced anticancer and biological activity, including examples such as Paclitaxel [20], plant-derived fatty acids [21], nigella sativa [22], and resveratrol [23]. Based on the provided information, the primary objective of the current investigation was to assess the anticancer effects of SES and/or SES-NS alone and in combination with EPI on SEC-bearing mice. Additionally, the study aimed to evaluate the ability of SES to mitigate various toxicities induced by EPI treatment.

## 2. Materials and Methods

### 2.1. Ethical Concerns

Ethical approval for the study was obtained from the Ethical Committee at the Faculty of Pharmacy, Damanhour University (Reference no.: 921PB23).

### 2.2. Reagents and Chemicals

SES 98% (Sigma-Aldrich, Catalog No.: S3003-5G) and EPI (Ebewe Company, Catalog No.: LG4092, Unterach am Attersee, Austria) were employed in the study. Solvents utilized for SES-NS (including sodium lauryl sulfate, poly-vinyl alcohol (PVA), acetone, and Tween 80) were procured from Sigma-Aldrich, Dorset, UK. The EST was obtained from the Medical Research Institute, Alexandria University, Alexandria, Egypt. All other chemicals utilized in the study were of analytical grade and sourced from commercial suppliers.

### 2.3. Preparation of SES Acid-Loaded Nanosuspension (SES-NS)

The SES-NSs were synthesized using a solvent evaporation antisolvent precipitation method employing probe sonication [24]. To prepare a 50 mg/mL stock solution, SES was dissolved in acetone. Tween 80 and SLS were explored as co-stabilizers, as outlined in Table 1. Utilizing probe sonication (QS4 system, NanoLab, Waltham, MA, USA), the drug solution was gradually added dropwise over one minute into the antisolvent stabilizer solution. Sonication was conducted for 15 min at an amplitude of 80% (of 125 W, 20 KHz), with 10 s pulses on and 5 s pulses off. To maintain the stabilizer solution’s temperature at 5 °C ± 3 °C, sonication was performed in an ice bath. Subsequently, under magnetic stirring and ambient temperature conditions, the drug dispersion was allowed to precipitate in nanosized form through solvent evaporation. The prepared formulations were stored in the refrigerator at 2–8 °C.

### 2.4. Particle Size and Zeta Potential Measurement

NS preparations were characterized for particle size, polydispersity index (PDI), and zeta potential analysis by dynamic light scattering using a Microtrac particle sizer and zeta potential analyzer (Microtrac Retsch Gmbh, Haan, Germany).

### 2.5. Drug Content Homogeneity (DCH%)

To evaluate the drug content homogeneity (DCH%) in SES-NS, an aliquot of 100 μL of the prepared NS was evaporated overnight to dryness [25,26]. After dissolving the residue in acetone, the mixture was then centrifuged for 15 min at 14,000 rpm. After that, samples were run through a 0.20 μm filter and subjected to spectrophotometric analysis at 294 nm using a UV Spectrophotometer T80 UV-Vis (PG Instruments Ltd., Lutterworth, UK) 1800. The following equation was used to estimate the DCH%:

$$DCH\%=\frac{Calculated\;drug\;amount}{Theoretical\;drug\;amount}\times 100$$

### 2.6. Stability of SES-NS

SES-NSs were stored at 4 °C for 2 months and subjected to stability study by monitoring any change in particle size, zeta potential, and DCH%.

### 2.7. Animals

Healthy, non-pregnant female Swiss albino mice weighing 25 g were provided by the Animal Care Unit of Alexandria University Medical Research Institute (Alexandria, Egypt). They were allowed to acclimate under standard animal house conditions. The mice were provided with ad libitum access to tap water and a standard pellet diet throughout the experimental period. Environmental conditions were maintained at a temperature of 22 °C ± 3 °C and a consistent relative humidity.

### 2.8. Tumor Induction

After extracting ascitic fluid from mice infected with Ehrlich ascites carcinoma (EAC), it was diluted in normal saline to create a suspension, with each 0.1 mL containing 2.5 × 10^6^ of EAC cells [27]. Viability of the cells was confirmed using a Trypan blue dye exclusion experiment [28]. On the first day of the experiment, 0.2 mL of the Ehrlich’s ascites carcinoma suspension was subcutaneously injected into the mammary fat pad on the upper left ventral side of mice. Tumor growth was confirmed ten days post-injection using a caliper, and tumor volume was calculated using the formula: Tumor Volume = length × width 2 × 0.52. Treatment commenced once solid tumors (50–100 mm^3^) became visibly evident, ten days post-inoculation.

### 2.9. Experimental Design

Thirty-five female Swiss albino mice were randomly divided into seven groups, each containing five mice. The treatment protocols were as follows:

⮚ Group 1 (Control): Healthy mice were intraperitoneally (i.p.) injected with normal saline daily for 21 days.
⮚ Group 2 (EST): Ehrlich carcinoma-bearing mice were injected (i.p.) with normal saline daily for 21 days [29].
⮚ Group 3 (SES): Ehrlich carcinoma-bearing mice were orally treated with SES (70 mg/kg/day, p.o.) daily for 21 days [30].
⮚ Group 4 (SES-NS): Ehrlich carcinoma-bearing mice were orally treated with SES-NS (10 mg/kg/day, p.o.) daily for 21 days [31].
⮚ Group 5 (EPI): Ehrlich carcinoma-bearing mice were injected (i.p.) with EPI weekly for 3 weeks (2.5 mg/kg/weekly, i.p.) [32].
⮚ Group 6 (SES + EPI): Ehrlich carcinoma-bearing mice were treated with a combination of SES (70 mg/kg/day, p.o.) daily for 21 days and EPI weekly for 3 weeks (2.5 mg/kg/weekly, i.p.).
⮚ Group 7 (SES-NS + EPI): Ehrlich carcinoma-bearing mice were orally treated with SES-NS daily (10 mg/kg/day, p.o.) for 21 days and EPI weekly for 3 weeks (2.5 mg/kg/weekly, i.p.).

At the end of the experimental work, animals were euthanized, and blood samples were collected using orbital sinus [33]. Serum was obtained from these samples by centrifugation and stored at −20 °C until analysis. Solid tumors were then excised and weighed after euthanizing the mice.

### 2.10. Tumor Assessment

Tumor growth was monitored using a digital caliper ten days after inoculation, and tumor weight was measured at the end of the experiment using a digital balance scale [28].

### 2.11. Estimation of Akt, Lc3-II, and Beclin1 Expressions Using qRT-PCR Techniques

An RNeasy extraction kit (Qiagen GmbH, Qiagen Strasse1, 40724 Hilden, Germany. Cat. No.: 169027065) was utilized to extract total RNA from tumor tissues according to the manufacturer’s protocol. SYBR Green and the Master mix (Thermo Fisher Scientific, Inc., Waltham, MA, USA) were used to create the cDNA from the isolated RNA following the manufacturer’s protocol. qRT-PCR was used to assess the expressions of the Akt, Lc3 II, and beclin1 genes using primers (Table 2). There were three runs of the experiments. For each sample, the 2^−ΔΔCT^ method [34] was utilized to determine the relative expression levels of Akt, LC3 II, and beclin1 in relation to β-actin.

### 2.12. Estimation of Akt and Lc3-II Using ELISA Technique

The levels of AKT and lc3-II in serum were determined using the AKT ELISA Kit (Catalog No.: MBS3807575) and Autophagy related protein LC3 B ELISA Kit (Catalog No.: MBS1600540), respectively.

### 2.13. Estimation of VEGF-2, Caspase-3 BAX, TAC, and MDA

The levels of vascular endothelial growth factor receptor-2 (VEGFR-2), caspase-3, BAX, total antioxidant capacity (TAC), and malondialdehyde (MDA) in serum were determined using specific ELISA kits. A Mouse VEGFR-2/KDR ELISA Kit (Catalog No.: EM1445) was used for VEGFR-2 measurement, Mouse Caspase-3 ELISA kit (Catalog No.: MBS733100) for caspase-3, BAX (Bcl-2 Associated X Protein) ELISA Kit (Catalog No.: MBS2512405), Mouse Total Antioxidant Capacity (TAC) ELISA kit (Catalog No.: MBS733680) for TAC, and Malondialdehyde Colorimetric Assay Kit (TBA Method) (Catalog No.: E-BC-K025-M) for MDA. Manufacturer’s instructions were strictly followed for each assay.

### 2.14. Estimation of TnI, CK-MB, Creatinine, and Urea

Serum levels of Troponin I (TnI), Creatine kinase-MB (CK-MB), creatinine, and urea were assessed using specific ELISA kits. A Mouse Troponin I ELISA Kit (Catalog No.: ab285235) was used for TnI measurement, Mouse CK-MB ELISA Kit (Catalog No.: NBP2-75312) for CK-MB, Mouse Creatinine Kit (Catalog No.: 80350) for creatinine, and urea nitrogen assay kit for urea.

### 2.15. Assessment of Serum ALT and AST Enzymes

Serum aspartate transaminase (AST) and alanine transaminase (ALT) levels were determined according to the method by Bergmeyer and Horder, 1980 [35].

### 2.16. Immunohistochemical Analysis

Thick paraffin slices (4 μm) were prepared, deparaffinized, rehydrated, and treated with 3% H_2_O_2_ for deactivation. After washing with PBS, they were blocked with 10% regular blocking serum for 60 min at room temperature. Subsequently, primary antibodies such as VEGF were applied overnight at 4 °C. After washing with PBS, the sections were incubated with biotin-conjugated secondary antibodies, followed by streptavidin-peroxidase conjugate. Visualization was performed using 3,3′-diaminobenzidine tetrahydrochloride (DAB), and Mayer’s hematoxylin was used for counterstaining. Immunostained slides were photographed, and quantitative histomorphometric analysis was performed using ImageJ software. Then, the primary antibody of VEGF (BioGenex, Fremont, CA, USA, catalog NO: AR483–5R) was incubated at 4 °C overnight, washed with PBS and incubated with biotin-conjugated goat anti-rabbit IgG antiserum (Histofine kit, Nichirei Corporation, Tsukiji, Chuo-ku, Tokyo, 104-8402, Japan) for 60 min. After washing with PBS, the sections were incubated with streptavidin–peroxidase conjugate (Histofine kit, Nichirei Corporation, Tsukiji, Chuo-ku, Tokyo, 104-8402, Japan) for 30 min. The streptavidin–biotin complex was visualized with 3,3′-diaminobenzidine tetrahydrochloride (DAB). Mayer’s hematoxylin was then used as a counterstain after the sections had been cleaned in distilled water. Stained slides were then photographed by a digital camera (Leica EC3, Leica, Wetzlar, Germany) for the quantitative histomorphometric analysis (10 random fields from each section [36]. The histomorphometric analysis was evaluated using ImageJ software (v1.46r, NIH, Bethesda, MD, USA) [37].

### 2.17. Histopathological Investigation

Tumors, livers, heart, and kidneys were surgically removed and fixed with 4% paraformaldehyde (PFA) dissolved in PBS for 48 h after being flushed with phosphate buffer saline (PBS, pH 7.4). The fixed specimens were then processed by the conventional paraffin embedding technique, cleared in three changes of xylene and melted paraffin, and ended by embedding in paraffin wax at 65 °C. Four µm thick sections were stained by Hematoxylin and Eosin (HE) [38].

### 2.18. Statistical Analysis

Using GraphPad Prism 3.0 (GraphPad Software, La Jolla, CA, USA), the data were statistically analyzed. One-way ANOVA analysis of variance (ANOVA) followed by the Tukey post hoc test was used for the analysis. The mean ± standard deviation (SD) is used to present the data. A statistically significant value was defined as a probability value (*p*-value) less than 0.05.

## 3. Results

### 3.1. Tumor Weight

**Figure 1:**
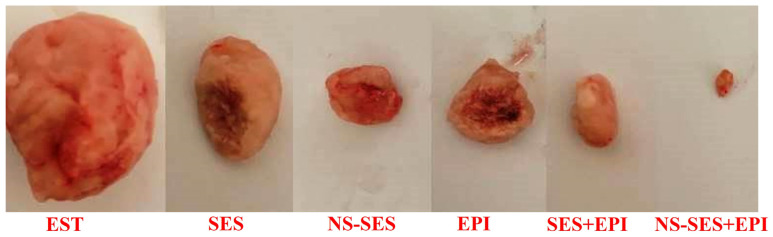
Tumor weight in all experimental groups. The abbreviations used are as follows: EST (Ehrlich solid tumor), SES (sesamol), SES-NS (nanosuspension of sesamol), EPI (Epirubicin), SES + EPI (sesamol + Epirubicin), and SES-NS + EPI (nanosuspension of sesamol + Epirubicin).

The tumor weight exhibited a decrease in all treatment groups. Notably, mice treated with SES-NS displayed a significantly reduced tumor weight (gm) (1.24 ± 0.36) (*p* < 0.001) compared to both the EST (3.788 ± 0.22) and SES (2.58 ± 0.47) groups. Remarkably, the combined treatment groups, SES + EPI (1.238 ± 0.61) and SES-NS + EPI (1.211 ± 0.54), demonstrated the most substantial reduction in tumor weight (Figure 1).

### 3.2. Particle Size and Zeta Potential Measurement

**Figure 2:**
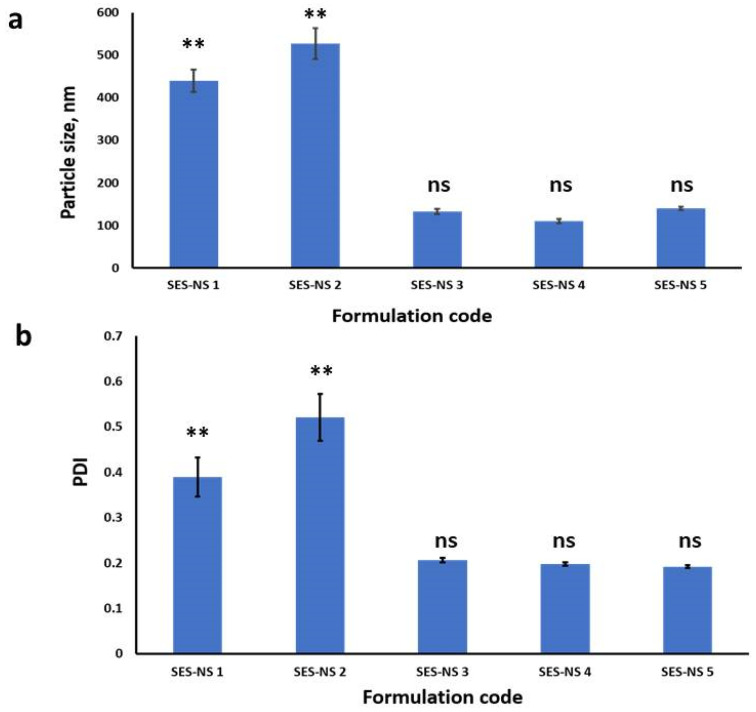
(**a**) Particle size distribution of SES-NS formulations. (**b**) PDI of SES-NS formulations. Means ± SD, *n* = 3, ** Significant, ns non-significant difference using one-way ANOVA and Tukey post hoc test.

Measurements of zeta potential and particle size were performed on the prepared SES-NS formulations. Different SES-NSs were prepared from different combinations of PVA, Tween 80 and SLS, according to Table 1. The obtained particle size distribution and PDI values are shown in Figure 2a,b. SES-NS 4 showed the smallest monomodal particle size of (110.23 ± 4.59) and of PDI (0.198 ± 0.004). The developed SES-NS 5 showed a negative zeta potential of (−28.46 ± 1.74 mV).

### 3.3. Drug Content Homogeneity (DCH%)

DCH% was assessed using the evaporation filtration technique, followed by spectrophotometric analysis. The prepared SES-NS 5 exhibited a marked drug loading of 96.34% ± 3.58.

### 3.4. Stability of SES-NSs

**Figure 3:**
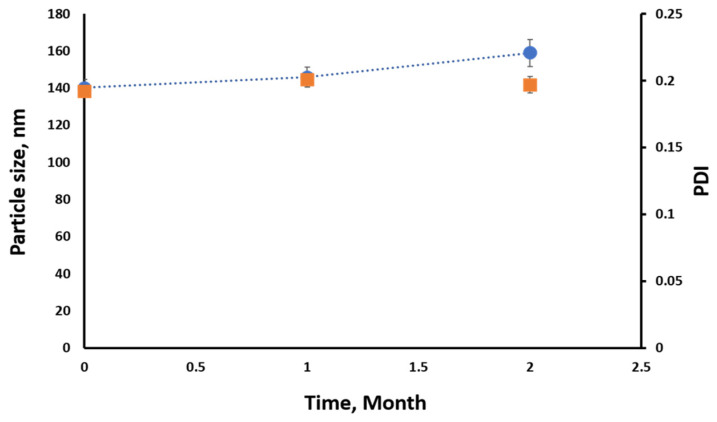
The particle size distribution and polydispersity index (PDI) values of SES-NS formulations during a two-month storage period at 4 °C. The data are presented as mean ± standard deviation (SD), with three samples analyzed at each time point.

The chosen SES-NS 5 formulation underwent a stability study lasting two months. During this period, the nanoparticle (NS) formulation was evaluated for changes in particle size, polydispersity index (PDI), zeta potential value, and drug loading percentage (DCH%). As depicted in Figure 3, SES-NS 5 maintained its uniform particle size distribution and PDI values consistently without significant deviation from the original formulations over the storage duration at 2–8 °C. Furthermore, SES-NS 5 retained its negative zeta potential at approximately −26.46 ± 0.946 mV, and its drug loading percentage remained stable at 93.79% ± 4.94.

### 3.5. Expression of AKT, Beclin1 and LC3

**Figure 4:**
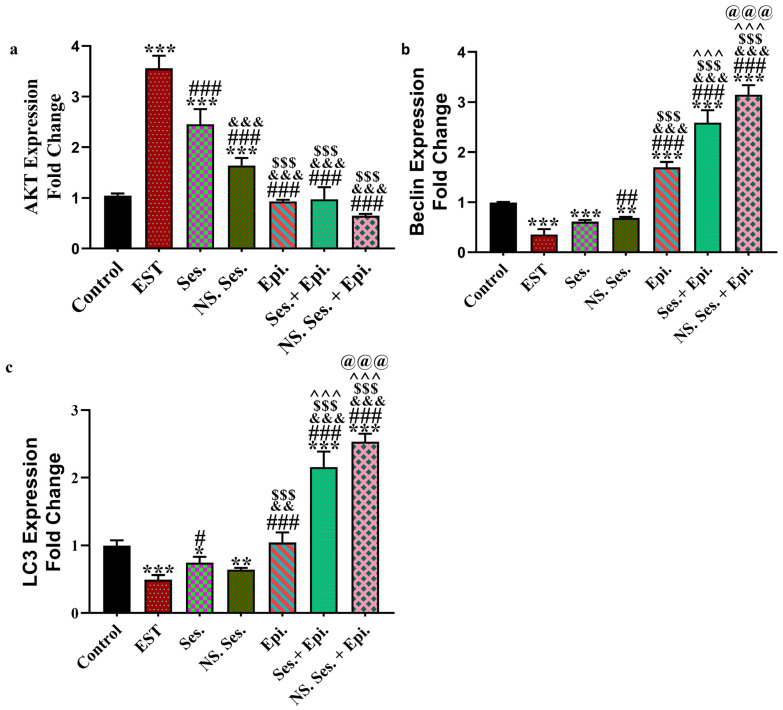
(**a**) mRNA expression of AKT in all studied groups, (**b**) mRNA expression of Beclin1 in all studied groups, and (**c**) mRNA expression of LC3 in all studied groups using qRt-PCR with specific primers. Data expressed as mean ± SD. *, #, &, $, ^, and @ indicate significant change from control, EST, SES, SES-NS, EPI, and SES + EPI respectively.

**Figure 5:**
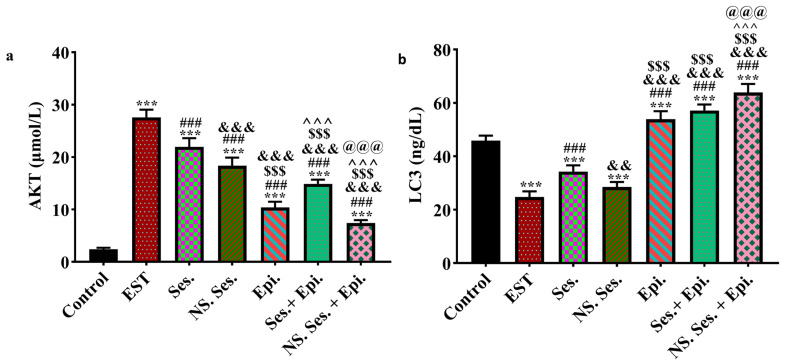
(**a**) Expression of AKT in all studied groups and (**b**) expression of LC3 in all studied groups using ELISA technique. Data expressed as mean ± SD. *, #, &, $, ^, and @ indicate significant change from control, EST, SES, SES-NS, EPI, and SES + EPI respectively.

Compared to the control group, the EST group exhibited a significant elevation in serum AKT levels. Conversely, groups treated with SES, SES-NS, and EPI revealed a noteworthy decrease in the AKT level when compared with the EST group. The combination treatment with SES + EPI and SES-NS + EPI showed the most significant decrease in AKT levels (Figure 4a and Figure 5a). Furthermore, the expression levels of Beclin1 and LC3 genes were significantly higher in the EST group compared to the control group. However, when compared to the EST and control groups, the combination therapy groups SES + EPI and SES-NS + EPI showed a significant increase in Beclin1 and LC3 levels (Figure 4b,c and Figure 5b).

### 3.6. Estimation of Caspase3, BAX, MDA, and TAC

**Figure 6:**
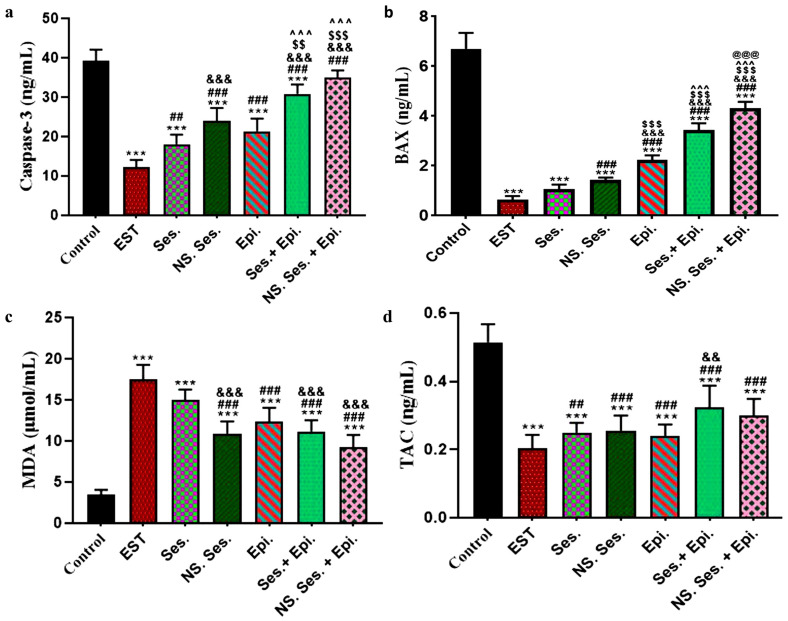
(**a**) Serum levels of caspase3 in all studied groups, (**b**) serum levels of BAX in all studied groups, (**c**) serum levels of MDA in all studied groups, and (**d**) serum levels of TAC in all studied groups. Data expressed as mean ± SD (*n* = 5). *, #, &, $, ^, and @ indicate significant change from control, EST, SES, SES-NS, EPI, and SES + EPI respectively.

In contrast to the control group, the EST group exhibited notably lower serum levels of caspase-3 and BAX. However, these levels increased in groups treated with SES, SES-NS, and EPI compared to the EST group. Furthermore, the combination therapy groups, SES + EPI and SES-NS + EPI, showed significantly higher caspase-3 and BAX levels compared to other groups (Figure 6a,b). Regarding malondialdehyde (MDA), the EST group displayed the highest level compared to the control group. Notably, groups treated with SES + EPI and SES-NS + EPI exhibited the most substantial reduction in the MDA serum level compared to the EST group (Figure 6c). Additionally, compared to the control group, the TAC level in the EST group significantly decreased. However, all other treated groups demonstrated a marked elevation in TAC serum level compared to the EST group (Figure 6d).

### 3.7. Serum Level of VEGFR2, TnI, and CK-MB

**Figure 7:**
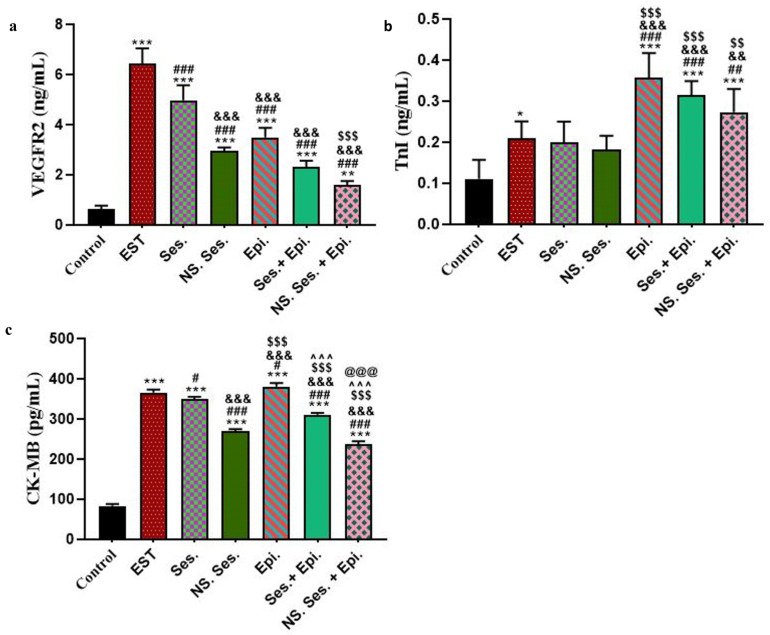
(**a**) Levels of VEGFR2 in all studied groups, (**b**) levels of TnI in all studied groups, and (**c**) levels of CK-MB in all studied groups. Data expressed as mean ± SD (*n* = 5). *, #, &, $, ^, and @ indicate significant change from control, EST, SES, SES-NS, EPI, and SES + EPI respectively.

The EST group exhibited the highest level of VEGFR2 compared to the control group. However, VEGFR2 levels decreased significantly in the SES-NS group compared to both the SES and EST groups. The most noteworthy decrease in VEGFR2 level was observed in the combination therapy groups, SES + EPI and SES-NS + EPI, compared to the EST group and their respective monotherapy groups (Figure 7a). There were no significant differences in TnI levels between the SES, SES-NS, and control groups. However, the EPI group displayed the highest serum TnI level compared to the control, EST, SES, and SES-NS groups. Remarkably, the combination therapy groups exhibited a significant reduction in TnI levels compared to the EPI-treated group (Figure 7b). In comparison to the control group, the EST group showed a considerably higher serum level of CK-MB. The EPI group exhibited the highest serum CK-MB level. Conversely, other treated groups with SES, SES-NS, and combined drugs demonstrated a reduction in CK-MB serum level compared to the EPI and EST groups, with the most substantial reduction observed in the SES-NS + EPI group (Figure 7c).

### 3.8. Serum ALT and AST Enzymes

The EPI group exhibited a considerable increase in ALT and AST values as compared to the treated groups. However, the combination therapy groups, SES + EPI and SES-NS + EPI, exhibited a significant decrease in ALT and AST levels compared to both the EST and EPI groups (Table 3).

### 3.9. Serum Creatinine and Urea

The EST group displayed an elevation in serum creatinine levels compared to the control group. While SES-NS showed a decrease in serum creatinine levels compared to the SES group, the EPI group revealed a significant elevation in serum creatinine levels compared to the control, EST, SES, and SES-NS groups. Remarkably, the combination therapy groups, SES + EPI and SES-NS + EPI, demonstrated a decrease in serum creatinine levels compared to the EPI-treated group (Table 3). Furthermore, urea serum levels markedly increased in the EPI group compared to the control, EST, SES, and SES-NS groups. However, the combination therapy groups, SES + EPI and SES-NS + EPI, exhibited decreased serum urea levels compared to the EPI-treated group (Table 3).

### 3.10. Immunohistochemical Findings

**Figure 8:**
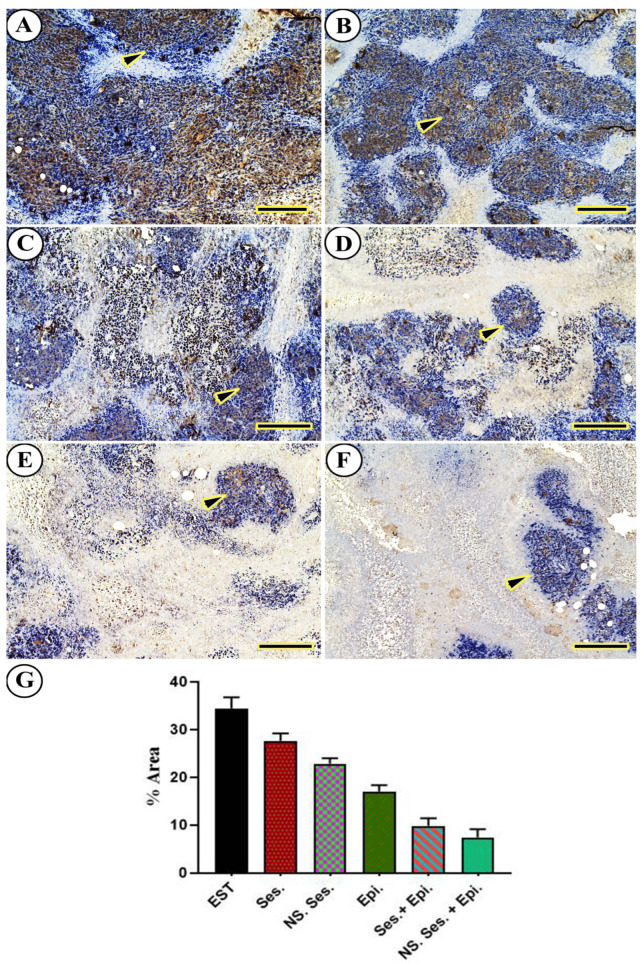
A representative photomicrograph illustrates VEGFR2 immunohistochemical expression in a mouse tumor from (**A**) EST, (**B**) SES, (**C**) SES-NS, (**D**) EPI, (**E**) SES + EPI, and (**F**) SES-NS + EPI. Arrowheads indicate positive immune expression. Scale bar = 100 µm. (**G**) One-way ANOVA was performed at *p* ≤ 0.05 and the data are presented as mean ± SE.

The expression of VEGFR2 was evaluated in excised mammary tumors. The EST group displayed the highest expression of VEGFR2 (Figure 8A). Conversely, EST mice treated with SES and NS-SES exhibited a reduced distribution of VEGFR2 compared to the EST group (Figure 8B,C). Furthermore, among all groups, VEGFR2 distribution was lowest in EST mice treated with EPI, SES + EPI, and SES-NS + EPI (Figure 8D–F). Comparatively, EST-treated mice demonstrated a noticeably higher expression of VEGFR2 compared to SES, SES-NS, EPI, SES + EPI, and SES-NS + EPI, as per the nonparametric quantitative analysis.

### 3.11. Histopathological Examination

**Figure 9:**
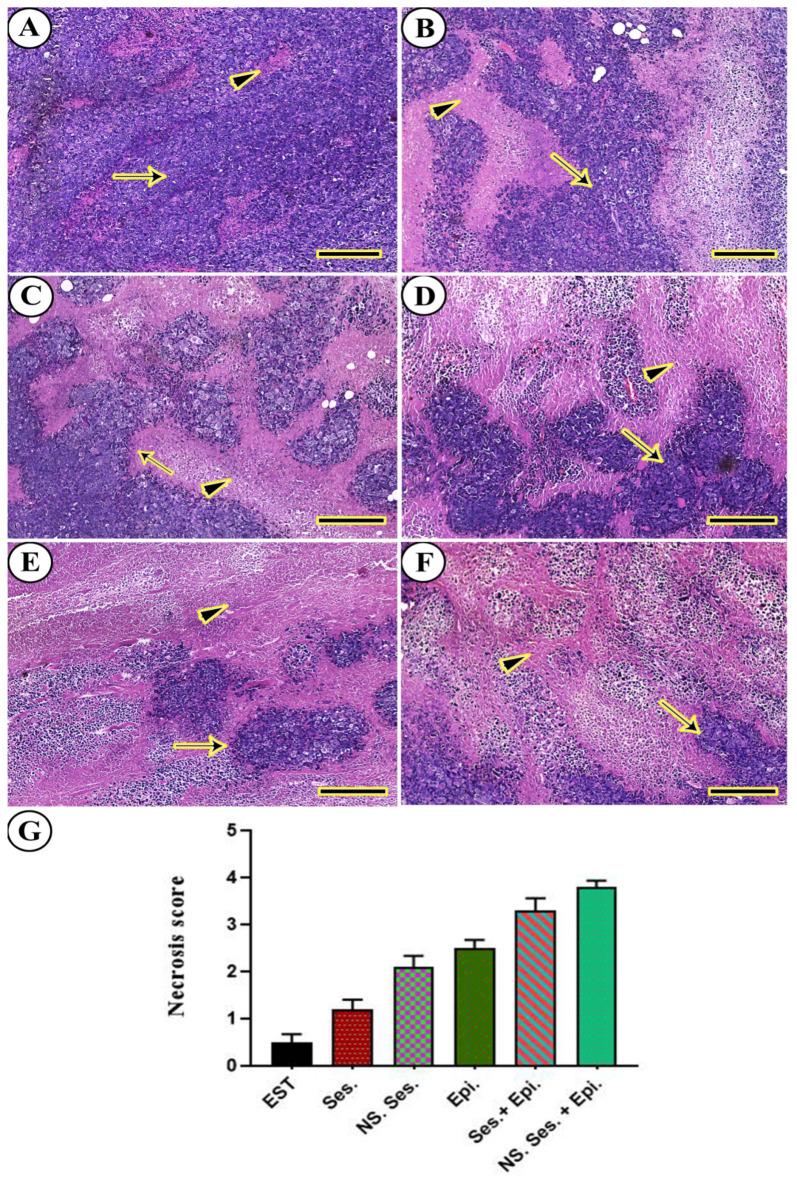
Histopathological analysis was conducted on solid mammary tumors excised from mice treated with various therapies. (**A**) EST. (**B**) SES. (**C**) SES-NS. (**D**) EPI. (**E**) SES + EPI. (**F**) SES-NS + EPI. Arrows indicate the neoplastic tumors and arrowheads indicate the necrotic areas with different stages. Scale bar = 100 µm. (**G**) H and E semi-quantitative scoring of tumor necrosis. One-way ANOVA was performed at *p* ≤ 0.05 and the data are presented as mean ± SE.

During histopathological examination, the solid mammary tumors removed from animals treated with EST, SES, SES-NS, EPI, SES + EPI, and SES-NS + EPI displayed confined nodules containing both viable and necrotic pleomorphic malignant cells with weak differentiation. The viable cancerous cells exhibited large hyperchromatic nuclei, noticeable nucleoli, anisokaryosis, and bipolar to multipolar mitotic division (Figure 9A–F). Furthermore, the semi-quantitative scoring of necrosis in each excised tumor revealed a significant increase in the necrotic area score in all treated groups compared to the EST group (Figure 9G).

**Figure 10:**
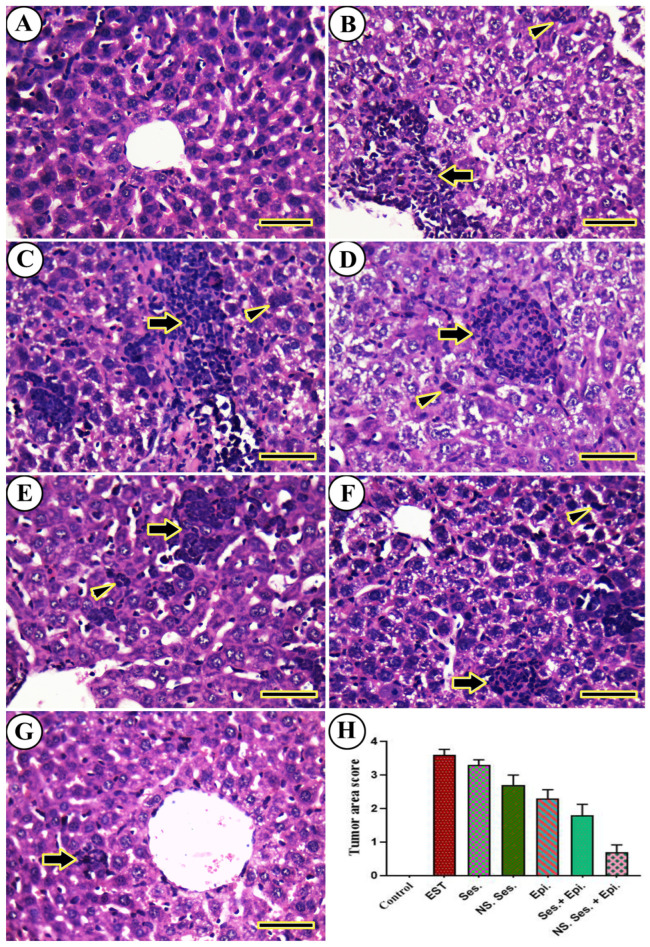
Representative photomicrographs for H&E-stained liver in mouse from (**A**) negative control, (**B**) EST, (**C**) SES, (**D**) SES-NS, (**E**) EPI, (**F**) SES + EPI and (**G**) SES-NS + EPI. Thick arrow: pleomorphic, hyperchromatic, metastatic tumor foci; arrowheads: invasion of sinusoids with tumor and Kupffer cells. Scale bar = 50 µm. (**H**) Semi-quantitative scoring of histopathological lesion score. One-way ANOVA was performed at *p* ≤ 0.05 and the data are presented as mean ± SE.

**Figure 11:**
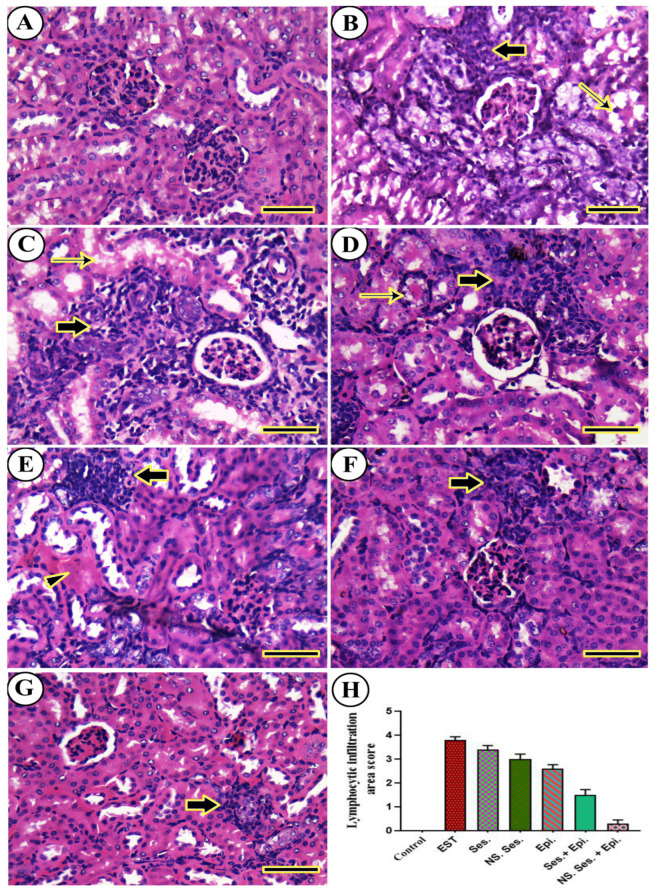
Representative photomicrographs for H&E-stained kidney in mouse from (**A**) negative control, (**B**) EST, (**C**) SES, (**D**) SES-NS, (**E**) EPI, (**F**) SES + EPI and (**G**) SES-NS + EPI. Thick arrow: periglomerular and perivascular inflammatory infiltration; thin arrow: degenerated tubules; arrowhead: intervascular congestion. Scale bar = 50 µm. (**H**) Semi-quantitative scoring of histopathological lesion score. One-way ANOVA was performed at *p* ≤ 0.05 and the data are presented as mean ± SE.

The histopathological examination of liver tissues is shown in Figure 10. As shown, in the negative control liver (Figure 10A), no tumor cells were observed. However, in the liver tissues of the EST group, metastatic tumor cells with pleomorphic and hyperchromatic features were detected (Figure 10B). Similar histopathological characteristics of malignant cells with varying degrees of invasion were observed in all treated animals. Mice treated with SES-NS + EPI displayed only a small number of aberrant hepatocyte mitotic figures (Figure 10C–G). Furthermore, the semi-quantitative scoring of metastatic tumors in each liver revealed a significant increase in the metastatic tumor area score in all treated mice compared to the EST group (Figure 10H). Figure 11 depicts the histopathology of kidney. As shown, in the untreated positive control mice, the kidneys did not display metastatic tumorous invasion. However, notable perivascular and periglomerular aggregations of long-term inflammatory cells, primarily lymphocytes, were observed. Additionally, atrophied glomeruli and modest necrotic and degenerative alterations in the renal tubular epithelium were present (Figure 11B). Animals treated with SES, SES-NS, EPI, SES + EPI, and SES-NS + EPI exhibited similar histologic characteristics, including varying levels of necrotic, degenerative, and inflammatory infiltration (Figure 11C–G). Furthermore, the semi-quantitative scoring of lymphocytic infiltration in each kidney revealed a significant increase in the lymphocytic infiltration area score in the SES, SES-NS, EPI, SES + EPI, and SES-NS + EPI groups compared to the EST group (Figure 11H).

**Figure 12:**
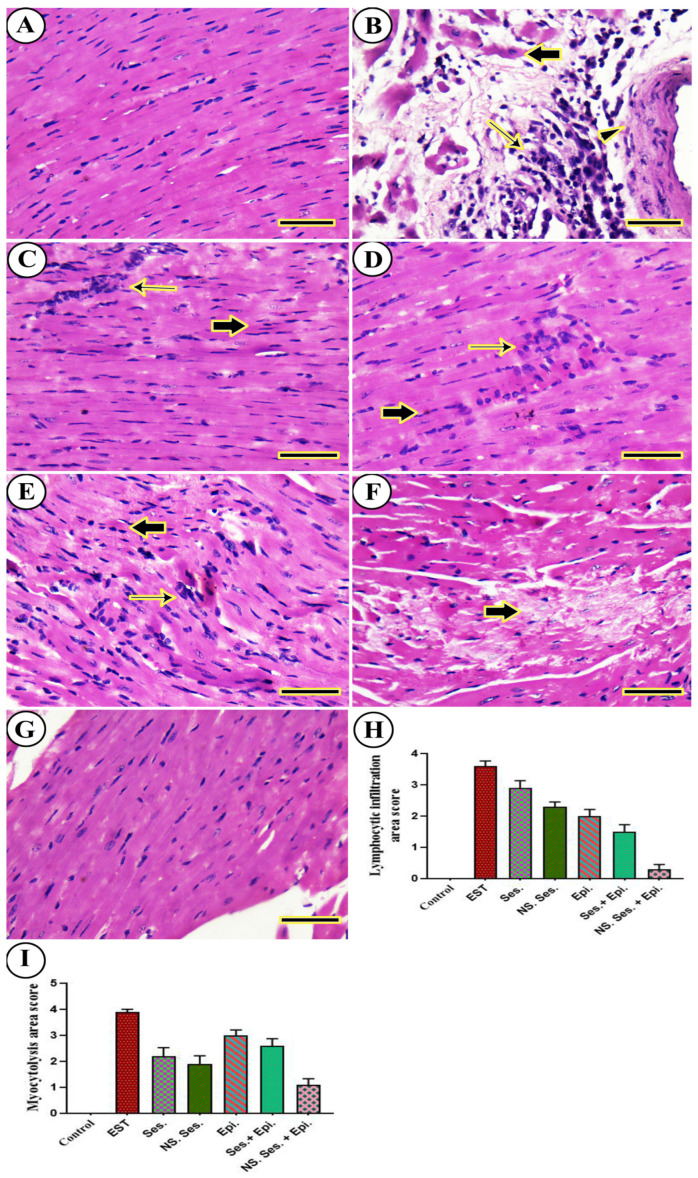
Representative photomicrographs for H&E-stained heart in mouse from (**A**) negative control, (**B**) EST, (**C**) SES, (**D**) SES-NS, (**E**) EPI, (**F**) SES + EPI and (**G**) SES-NS + EPI. Thick arrow: degeneration and lysis of cardiac myocytes; thin arrow: infiltration of mononuclear chronic inflammatory cells; arrowhead: degeneration in tunica media and adventitia of arterial wall. Scale bar = 50 µm. (**H**,**I**) Semi-quantitative scoring of histopathological lesion score. One-way ANOVA was performed at *p* ≤ 0.05 and the data are presented as mean ± SE.

The histopathology of the heart of positive control mice displayed hyalinization and cytolysis of myofibers, perivascular infiltration of mononuclear chronic inflammatory cells, and degeneration in the tunica media and adventitia of the arterial wall (Figure 12B). However, the SES and SES-NS groups revealed moderate interfibrillar infiltration of pleomorphic mononuclear chronic inflammatory cells with moderate degeneration of cardiac myocytes (Figure 12C,D). The EPI group exhibited disruption and degeneration of myocardial bundles along with mononuclear inflammatory infiltration (Figure 12E). In contrast, the SES + EPI group displayed myocytolysis and interfibrillar edema with minimal interfibrillar infiltration of pleomorphic mononuclear chronic inflammatory cells (Figure 12F). Notably, the SES-NS + EPI group exhibited a cardiac architecture like the negative control (Figure 12G). Furthermore, the semi-quantitative scoring of lymphocytic infiltration and myocytolysis in each heart revealed a significant increase in the lymphocytic infiltration and myocytolysis area score in all treated mice compared to the EST group (Figure 12H).

## 4. Discussion

As mentioned above, BC stands out as the most frequently diagnosed cancer, contributing significantly to the high mortality rates recorded worldwide [39]. Treatment options for BC encompass a range of strategies, including chemotherapy, radiation therapy, and surgery. Among these, chemotherapy remains a cornerstone in the treatment of cancer, including breast cancer. Epirubicin (EPI) is one of the chemotherapeutic agents commonly employed for treating breast cancer. However, its indiscriminate toxicity towards both normal and cancerous cells, coupled with the development of resistance by cancer cells, compromises its efficacy [40]. Therefore, numerous investigations have directed significant attention towards addressing these challenges by exploring the potential of natural substances, either independently or in conjunction with chemotherapy medications [41]. As mentioned above, NS represents a biphasic drug dispersion comprising crystalline particles stabilized by surfactants and dissolved in a suitable aqueous medium. Fundamentally, NSs address issues related to the low bioavailability and solubility of hydrophobic substances [42], as the formed nanosized-particles produce higher surface areas, leading to greater dissolution rates and subsequently rising the drug’s bioavailability [43]. As a particulate carrier-free system, NS provides strong potential for sustained release, higher efficacy, decreased toxicity, and lower first-pass metabolism [43]. Therefore, NS was chosen as a viable nano-drug delivery system to enhance SES’s anticancer efficacy.

Considering the formulation aspects, NS preparation depends on the incorporation of surfactants and stabilizers with the minimal use of organic solvents. Surfactants are essential for the formulation of NSs to reduce the interfacial tension through wetting and/or deflocculating mechanisms [44]. Stabilizers, such as polymers (PVA) and polysorbates (tweens/spans), increase the wettability of drug nanocrystals and provide steric hinderance and/or ionic barriers to prevent Ostwald’s ripening and agglomeration of NS particles. Thus, stabilizers are important to maintain homogeneity of particle size and physical stability of NSs [42]. It is worth mentioning that the selected method of preparation, solvent evaporation antisolvent precipitation combined with probe sonication, eliminated the risk of residual solvent hazards in the final NS preparation.

Particle size distribution and PDI are the most vital parameters of nanosuspensions that reflect the drug’s solubility, stability, and pharmacological activities [44]. In the current work, various SES-NSs were prepared from different combinations of PVA, Tween 80, and SLS. The combination of SLS:Tween 80:PVA (1:0.5:0.5) in SES-NS 4 succeeded in producing a homogenous NS with the smallest monomodal particle size (110.23 ± 4.59, PDI 0.198 ± 0.004). A proper explanation could be that the combination of polymeric stabilizer (PVA), non-ionic surfactant (Tween 80), and ionic surfactant (SLS) confers a protective coating for the surface of the particles elucidating a dual role: preventing crystal growth and reducing particle size. Both electrostatic and steric mechanisms complemented each other [43]. Further increase in SES concentration from 2 to 7 mg/mL did not significantly increase the particle size in SES-NS 5. Therefore, SES-NS 5 was selected as the optimum formulation with the higher drug loading.

The zeta potential value provides an estimation of the electric double layer around the charged particles [45]. The electric charge developed on a particle surface initiates electrostatic repulsion between the nanoparticles and hinders particles’ aggregation and precipitation. Therefore, zeta potential is one of the important factors affecting nanocarriers’ stability, entrapment efficiency, and interactions with biological systems in vivo [46,47,48]. It is worth mentioning that in electrostatic stabilization combined with steric stabilization (by using appropriate polymers), zeta potential of 20 mV could be sufficient to prevent aggregation and precipitation of drug particles [42]. Thus, the obtained zeta potential of SES-NS was considered satisfactory.

Concerning drug content homogeneity (DCH%), being a carrier-free system, NSs provide higher loading capacity compared to polymer- and lipid-based nanocarriers due to the high mass per volume ratios for NSs. Furthermore, NSs maintain the drug particles in a favorable nanosized crystalline state sufficient for enhancing solubility and overcoming delivery issues without the need to actually dissolve them [43]. In agreement with the aforementioned advantages, the prepared SES-NS 5 achieved a high drug loading of 96.34% ± 3.58.

Stability of nano-formulations ensures their reliable safety and efficiency. Therefore, the stability of nanosuspensions in drug delivery is a very critical aspect of this technology [42]. The prepared SES-NS 5 maintained its monomodal particle size distribution and PDI values without significant differences from fresh formulations throughout 2 months of storage at 2–8 °C. Furthermore, SES-NS 5 kept its negative zeta potential at −26.46 ± 0.946 mV and its DCH% at 93.79% ± 4.94. These results indicated long-term stability of the developed SES-NS 5. A proper explanation is that the dense solid state in the formulated NS increased resistance to oxidation and hydrolysis and raised physical stability to settling. Interestingly, the developed NS provided a synergetic protection through polymeric, ionic, and neutral stabilizers. Simply, this combination minimized self-repulsion of the charged surfactant molecules, permitted greater surface coverage, and eventually allowed for closer packing [43]. Therefore, SES-NS 5 provided promising potential to be used for further in vivo studies.

In the present study, we investigated the anticancer potential of SES in mice bearing Ehrlich solid tumors (ESTs), both when administered alone and in combination with EPI. Furthermore, we assessed SES’s capacity to mitigate the toxicities induced by EPI. Additionally, we compared the benefits of using a nano-formulation of SES with a conventional formulation. According to the findings of the current study, mice that were subcutaneously inoculated with Ehrlich ascites carcinoma (EAC) developed solid tumors within 10 days of inoculation. This observation aligns with previous studies conducted using the same model [49]. Moreover, EST-bearing mice treated with SES for 21 days exhibited a reduction in tumor size and weight, highlighting the inhibitory effects of SES on malignant cell development and its potential as an anticancer agent [50]. Similarly, groups treated with EPI showed a significant decrease in tumor weight and size, confirming the antitumor efficacy of EPI [51]. Combination therapy groups demonstrated a notable reduction in tumor weight and size, indicating an enhanced anticancer activity of EPI while mitigating its side effects, which aligns with the objective of our study. The nano-formulation of SES significantly reduced tumor size and weight either alone or in combination with EPI, underscoring the enhanced therapeutic effects and pharmacological actions of nanomedicines [18]. Our study elucidated various pathways involved in inducing apoptosis, inhibiting tumor growth and proliferation, promoting autophagy, restraining angiogenesis, and reducing oxidative stress.

Apoptosis is vital for the development and survival of living organisms, representing a programmed suicidal process in which specific enzymes are activated within cells, leading to the dissolution of nuclear and cytoplasmic protein components. Dysregulation of this apoptotic pathway can contribute to various disorders, including cancer, neurodegenerative diseases, and autoimmune conditions [52]. Caspase-3 plays a crucial role in the apoptotic death receptor pathway, and reduced caspase-3 activity has been documented in cases of inflammation and advanced cancer stages [53,54]. In the current research, treated groups exhibited enhanced apoptosis, as evidenced by elevated expression of apoptotic caspase-3 and BAX levels. This finding is consistent with an earlier study which reported that SES, either alone or in combination with DOX, upregulated death receptors Fas. This activation led to the formation of the death-inducing signaling complex (DISC) through Fas-associated-death domain protein (FADD) and pro-caspase-8, subsequently activating caspase-8 and caspase-3 [30]. Additionally, SES treatment can reduce Bcl-2 gene expression and stimulate Bcl-2-associated X protein (BAX) by tBid promoting the permeabilization of the outer membrane of the mitochondria and the release of cytochrome c, resulting in the activation of the intrinsic apoptotic pathway [30]. Moreover, SES could induce apoptosis and inhibit cell proliferation by blocking the pi3k/AKT pathway, an essential signal transduction pathway [55]. It should be noted that AKT plays a role in maintaining mitochondrial function and energy production. In our study, treated groups exhibited decreased cell growth and proliferation due to AKT inactivation, which is consistent with previous findings demonstrating SES-induced intrinsic and extrinsic apoptosis, cell cycle arrest, and reduced cell viability through oxidative stress induction and AKT inactivation [56].

Autophagy is a cellular process that mitigates oxidative stress and protects cells from damage. It is triggered in response to nutrient deficiencies and anticancer medications to maintain cellular survival [57]. Beclin-1 and LC3 are key mediators of autophagy, with Beclin-1 being essential for autophagosome formation [57,58]. In the present study, Beclin-1 and LC3 levels were significantly lower in the EST group compared to the control group, while their levels were markedly increased in SES-treated groups and combination therapy groups. This finding is consistent with a previous study which demonstrated that SES increased Beclin-1 and LC3 expression, thereby stimulating autophagy in esophageal squamous cell carcinoma (ESCC) Eca109 cells [59].

Angiogenesis is a complex process by which new blood vessels are formed from preexisting ones, involving interactions between endothelial cells [60]. In this respect, vascular endothelial growth factor (VEGF) is considered an important angiogenic growth factor that regulates angiogenesis through the receptor tyrosine kinase VEGF receptor (VEGFR) [61]. Phosphorylated VEGFR2 triggers several cellular reactions and starts downstream signaling pathways involved in angiogenesis [62]. In the current research, the EST group displayed a significant elevation in VEGFR2 levels compared to the control group. Conversely, VEGFR2 levels in the SES-treated groups, whether administered alone or in combination with EPI, were notably lower than those in the EST group. These findings are consistent with a prior study that demonstrated that SES downregulated the lipopolysaccharide (LPS)-mediated overexpression of VEGFR2 [63].

It should be stressed that oxidative stress and reactive oxygen species (ROS) are known as major contributors to cancer growth. In our study, the serum levels of MDA exhibited a significant increase in the EST group compared to the control group, indicating elevated lipid peroxidation and a decrease in total antioxidant capacity (TAC), reflecting impairment in the antioxidant mechanism. However, SES-treated groups, whether administered alone or in combination with EPI, showed a significant decrease in MDA levels and a significant increase in TAC levels. This finding is consistent with a previous study demonstrating that SES mitigates oxidative stress in lung cells [64]. Furthermore, another study reported that SES, either alone or in combination with DOX, could mitigate oxidative stress in BC [30].

As mentioned, EPI is commonly used in the treatment of breast cancer. However, its toxicities often limit its clinical utility [51]. Consequently, numerous efforts have been made to alleviate its side effects. In our study, EPI treatment led to a significant increase in CK-MB levels compared to the control group. Additionally, histopathological examination revealed disruption and degeneration of myocardial bundles alongside mononuclear inflammatory infiltration, confirming the cardiotoxic effects induced by Epirubicin. However, combination therapy with SES + EPI resulted in a marked reduction in CK-MB levels. These findings align with a previous study demonstrating a reduction in CK-MB levels in breast cancer treatment with SES + DOX combination therapy [30]. Furthermore, a significant decrease in TnI levels was observed, consistent with prior research demonstrating a reduction in TnI and CK-MB levels and exerting a protective effect against isoproterenol-induced myocardial infarction [65]. These findings reflect the cardioprotective effects of SES against the cardiotoxic effects of chemotherapy.

Furthermore, serum urea and creatinine levels were significantly higher in the EPI-treated group compared to the control group. The histopathological examination illustrated inflammatory infiltration, degenerative alterations, and necrotic changes in kidney tissues, indicating EPI-induced nephrotoxicity. Conversely, groups treated with combination therapy demonstrated a notable decrease in the blood levels of creatinine and urea. These findings reveal the nephroprotective benefits of SES, which are consistent with a previous study demonstrating that SES reduced serum creatinine and blood urea nitrogen (BUN) levels in cisplatin-induced nephrotoxicity [66].

Moreover, elevated serum liver enzymes (ALT, AST) were documented in the EPI-treated group. Meanwhile, the histopathological examination confirmed the presence of malignant cells with various degrees of invasion, indicative of the hepatotoxic effects of EPI. In contrast, groups treated with combination therapy, particularly the SES-NS + EPI group, exhibited a significant reduction in the serum levels of ALT and AST. These results align with previous research that documented the hepatoprotective effects of SES-loaded solid lipid nanoparticles on rats with sub-chronic hepatotoxicity induced by carbon tetrachloride (CCl4) [67].

## 5. Conclusions

Based on the findings described above, the current study demonstrates that SES exhibits the potential to inhibit tumor growth in the EST mice model. It presents a promising avenue for cancer treatment through various mechanisms, including the reduction in oxidative stress, inhibition of angiogenesis, and induction of autophagy and apoptosis. Particularly when combined with EPI, SES enhances the effectiveness of cancer treatment compared to when EPI or SES is used alone. Notably, SES, especially in its nanosuspension form (SES-NS), effectively mitigates the toxicities induced by EPI while enhancing its anticancer effects. Further research is recommended to elucidate the mechanistic pathways underlying these anticancer effects of SES.

## Figures and Tables

**Table 1:** Composition of blank and loaded NLC formulations.

	Formulation	SM-NS 1	SM-NS 2	SM-NS 3	SM-NS 4	SM-NS 5
Composition						
SES (mg/mL)		2	2	2	2	7
SLS (% *w*/*w*)		-------	-------	1	1	1
Tween 80 (% *w*/*w*)		1	0.5	0.3	0.5	0.5

**Table 2:** Primer sequence used for qRT-PCR.

Primer Sequence	Gene (Amplicon Size)
F: CATTGCTGACAGGATGCAGA R: CTGCTGGAAGGTGGACAGTGA	β-actin (139 bp)
F: AGAAGAGACGATGGACTTCCG R: TCAAACTCGTTCATGGTCACAC	AKT (111 bp)
F: CGCTTGCAGCTCAATGCTAAC R: CTCGTACACTTCGGAGATGGG	LC3-II (93 bp)
F: ATGGAGGGGTCTAAGGCGTC R: TGGGCTGTGGTAAGTAATGGA	Beclin1 (149 bp)

The primers for β-actin, AKT, LC3 II, and Beclin1 were designed using the NCBI reference sequence.

**Table 3:** Estimation of ALT, AST, urea, and creatinine.

Variables	Studied Groups						
	Control.	EST.	SES.	SES-NS.	EPI.	SES. + EPI.	SES-NS. + EPI.
	*n* = 5	*n* = 5	*n* = 5	*n* = 5	*n* = 5	*n* = 5	*n* = 5
ALT (IU/L)	39.27 ± 2.81 35.45–42.19	78.88 ± 3.70	70.66 ± 3.28	54.41 ± 3.27	72.7 ± 2.21	52.34 ± 3.22	42.38 ± 4.06
Mean		74.34–83.28	66.59–74.4	49.77–58.5	69.49–75.45	48.28–56.86	37.3–47.30
Min.–Max.		*p* < 0.001	*p* < 0.001	*p* < 0.001	*p* < 0.001	*p* < 0.001	*p* < 0.001
AST (IU/L)	31.24 ± 4.28 26.37–35.97	96.29 ± 4.36	73.36 ± 5.57	69.11 ± 6.00	74.42 ± 3.414	65.02 ± 6.232	46.81 ± 2.997
Mean		91.21 101.2	65.67 79.87	61.38 77.40	71.83–80.28	55.1–71.21	43.29–50.39
Min.–Max.		*p* < 0.001	*p* < 0.001	*p* < 0.001	*p* < 0.001	*p* < 0.001	*p* = 0.004
Urea (mg/dL)	9.026 ± 1.08 7.552–10.19	12.1 ± 2.24	11.01 ± 1.70	10.47 ± 1.56	21.11 ± 2.41	19.64 ± 1.64	17.22 ± 0.79
Mean		9.198 15.29	8.908 13.28	8.266 12.08	18.21–24.4	17.12–21.3	16.23–18.28
Min.–Max.		*p* = 0.1032	*p* = 0.5423	*p* = 0.8333	*p* < 0.001	*p* < 0.001	*p* < 0.001
Creatinine (mg/dL)	0.1086 ± 0.04 0.076–0.156	0.1474 ± 0.03	0.1176 ± 0.01	0.1028 ± 0.01	0.2242 ± 0.02	0.1966 ± 0.04	0.1564 ± 0.02
Mean		0.124–0.177	0.104–0.141	0.094–0.112	0.199–0.249	0.136–0.229	0.135–0.19
Min.–Max		*p* = 0.2023	*p* = 0.997	*p* = 0.9998	*p* < 0.001	*p* < 0.001	*p* = 0.0636

Data are presented as mean ± SD (Standard Deviation), ALT: Alanine transaminase, AST: Aspartate transaminase, EST.: Ehrlich solid tumor, SES.: sesamol, SES-NS.: nanosuspension of sesamol, EPI.: Epirubicin, SES. + EPI.: sesamol + Epirubicin, SES-NS. +EPI.: nanosuspension of sesamol+ Epirubicin, *n*: number of animals per group.

## Data Availability

The data that support the findings of this study are available on request from the corresponding author.
